# Adverse Events and Clinical Correlates in Asian Patients with Atrial Fibrillation and Diabetes Mellitus: A Report from Asia Pacific Heart Rhythm Society Atrial Fibrillation Registry

**DOI:** 10.3390/jcm13051274

**Published:** 2024-02-23

**Authors:** Tommaso Bucci, Katarzyna Nabrdalik, Alena Shantsila, Giulio Francesco Romiti, Wee-Siong Teo, Hyung-Wook Park, Wataru Shimizu, Hung-Fat Tse, Marco Proietti, Tze-Fan Chao, Gregory Y. H. Lip, Asia-Pacific Heart Rhythm Society Atrial Fibrillation Registry Investigators

**Affiliations:** 1 Liverpool Centre of Cardiovascular Science at University of Liverpool, Liverpool John Moores University and Liverpool Heart & Chest Hospital, Liverpool L7 8TX, UKknabrdalik@sum.edu.pl (K.N.); s.shantsila@liverpool.ac.uk (A.S.); giuliofrancesco.romiti@uniroma1.it (G.F.R.); 2 Department of General and Specialized Surgery, Sapienza University of Rome, 00185 Rome, Italy; 3 Department of Internal Diseases, Diabetology and Nephrology in Zabrze, Medical University of Silesia in Katowice, 40-055 Katowice, Poland; 4 Department of Translational and Precision Medicine, Sapienza University of Rome, 00185 Rome, Italy; 5 Department of Cardiology, National Heart Centre, Singapore 610041, Singapore; wswkiliteo@gmail.com; 6 Department of Cardiovascular Medicine, Chonnam National University Hospital, Gwangju 61469, Republic of Korea; mdhwp@chonnam.ac.kr; 7 Department of Cardiovascular Medicine, Nippon Medical School, Tokyo 113-8602, Japan; wshimizu@nms.ac.jp; 8 Division of Cardiology, Department of Medicine, School of Clinical Medicine, Queen Mary Hospital, The University of Hong Kong, Hong Kong SAR 999077, China; hftse@hku.hk; 9 Department of Clinical Sciences and Community Health, University of Milan, 20122 Milan, Italy; marco.proietti@unimi.it; 10 Division of Subacute Care, IRCCS Institute Clinici Scientifici Maugeri, 20138 Milan, Italy; 11 Institute of Clinical Medicine, Cardiovascular Research Center, National Yang Ming Chiao Tung University, Taipei 112, Taiwan; 12 Division of Cardiology, Department of Medicine, Taipei Veterans General Hospital, Taipei 112, Taiwan; 13 Danish Center for Health Services Research, Department of Clinical Medicine, Aalborg University, 9220 Aalborg, Denmark

**Keywords:** atrial fibrillation, Asians, diabetes, major bleedings, cardiovascular events

## Abstract

**Aims.** To evaluate the adverse events (and its clinical correlates) in a large prospective cohort of Asian patients with atrial fibrillation (AF) and diabetes mellitus (DM). **Material and Methods.** We recruited patients with atrial fibrillation (AF) from the Asia-Pacific Heart Rhythm Society (APHRS) AF Registry and included those for whom the diabetic mellitus (DM) status was known. We used Cox-regression analysis to assess the 1-year risk of all-cause death, thromboembolic events, acute coronary syndrome, heart failure and major bleeding. **Results.** Of 4058 patients (mean age 68.5 ± 11.8 years; 34.4% females) considered for this analysis, 999 (24.6%) had DM (age 71 ± 11 years, 36.4% females). Patients with DM had higher mean CHA_2_DS_2_-VASc (2.3 ± 1.6 vs. 4.0 ± 1.5, *p* < 0.001) and HAS-BLED (1.3 ± 1.0 vs. 1.7 ± 1.1, *p* < 0.001) risk scores and were less treated with rhythm control strategies compared to patients without DM (18.7% vs. 22.0%). After 1-year of follow-up, patients with DM had higher incidence of all-cause death (4.9% vs. 2.3%, *p* < 0.001), cardiovascular death (1.3% vs. 0.4%, *p* = 0.003), and major bleeding (1.8% vs. 0.9%, *p* = 0.002) compared to those without DM. On Cox regression analysis, adjusted for age, sex, heart failure, coronary and peripheral artery diseases and previous thromboembolic event, DM was independently associated with a higher risk of all-cause death (HR 1.48, 95% CI 1.00–2.19), cardiovascular death (HR 2.33, 95% CI 1.01–5.40), and major bleeding (HR 1.91, 95% 1.01–3.60). On interaction analysis, the impact of DM in determining the risk of all-cause death was greater in young than in older patients (*p* int = 0.010). **Conclusions.** Given the high rates of adverse outcomes in these Asian AF patients with DM, efforts to optimize the management approach of these high-risk patients in a holistic or integrated care approach are needed.

## 1. Introduction

Type 2 diabetes mellitus (DM) and atrial fibrillation (AF) are two commonly coexisting diseases with a global prevalence of almost 40 million for AF and 537 million for DM [1]. South-East Asia is inhabited by over a quarter (1.97 billion people) of the world’s population and by the year 2045, the number of adults with DM in South-East Asia is predicted to grow to 152 million which translates into 69% increase, exceeding the predicted population increase of 13% in Europe and of 24% in North America [2].

AF is the most common sustained cardiac arrhythmia worldwide and DM itself is a strong and independent risk factor for AF and AF-related complications [3]. With the increasing burden of DM, there is also an increase in the prevalence of AF and the projections for the future decades are also pessimistic with the AF prevalence increase in Taiwan from 1.5% in year 2011 to 4.0% in the year 2050; and in South Korea from 1.5% in 2015 to 5.8% in the year 2060 [4,5]. 

DM may have different demographic and clinical features in patients of Asian ancestry when compared with those from Western countries: DM in Asian patients develops at a younger age and at a lower body mass index (BMI), and leads to a higher risk of diabetes complications and premature death [6,7]; moreover, compared to patients from Europe and the United States, those from East-Asia present different genetic and socioeconomic backgrounds and different DM treatment modalities that can influence the clinical course of diabetes and diabetes-related complications [8].

Given the obvious ethnic differences in relation to DM, we aimed to focus on the clinical course of contemporary Asian patients with AF and DM who were enrolled in the Asia-Pacific Heart Rhythm Society (APHRS) AF-registry. We particularly focused on adverse events, rhythm control versus rate control, and quality of life of participating AF patients with DM.

## 2. Materials and Methods

For this analysis, we used data from a registry of consecutive non-valvular AF patients who referred to in- and outpatients cardiology consultation in 52 centers from five Asian geographical areas (Hong Kong, Japan, Taiwan, Singapore, Korea) under the Asia-Pacific Heart Rhythm Society (APHRS). Methods and protocol for this study was adopted from EURObservational Research Programme on Atrial Fibrillation (EURO-AF) Long Term General Registry [9]. This was a prospective, observational, multicenter study. The registry was started in 2015 and completed in 2017. 

Inclusion criteria were age ≥18 years and an electrocardiogram (ECG) confirmed diagnosis of AF (12-lead ECG, 24-h ECG Holter, or other electrocardiographic documentation) within the 12 months prior to enrolment. Exclusion criteria were lack of ECG attesting AF and the presence of atrial flutter. All eligible patients signed a written informed consent for participation in the registry according to the Declaration of Helsinki. At the baseline, investigators recorded patient’s comorbidities and medical treatments using a standardized electronic case report form. In this study, we considered the history of the following diseases: DM, chronic obstructive pulmonary disease, hypertension, coronary and peripheral artery disease, heart failure, dyslipidemia, history of stroke or transient ischemic attack, major bleeding, chronic kidney disease (CKD), active cancer, and dementia; and the use of the following medications: angiotensin-converting enzyme inhibitors, angiotensin receptor blockers, beta-blockers, statins, oral antidiabetics, insulin, digoxin, aldosterone blockers, calcium channel blockers, proton pump inhibitors, oral anticoagulants (OAC), antiplatelets, and antiarrhythmics (amiodarone, flecainide, propafenone, dronedarone, and sotalol). After baseline assessment, a 1-year follow-up was performed by the local cardiologist investigator. The study protocol was approved by the local ethics committees for the different countries (and in some countries, multiple sites) and was registered on ClinicalTrials.gov (NCT04807049).

### 2.1. Clinical Scores

The CHA_2_DS_2_-VASc score was calculated as follows: congestive heart failure (1 point); hypertension (1 point); age 65–74 (1 point) and ≥75 years (2 points); diabetes (1 point); stroke (2 points); vascular disease (1 point); and female sex category (1 point) [10].

HAS-BLED score was calculated as follows: uncontrolled hypertension (1 point), abnormal renal or liver function (defined as dialysis, renal transplant, serum creatinine >200 µmol/L for the former and liver cirrhosis, bilirubin > 2 × upper limit of normal, aspartate transaminase (AST)/alanine transaminase (ALT)/alkaline phosphatase (ALP) > 3 × upper limit of normal for the latter, 1 point each); history of stroke (1 point); history of bleeding (1 point); labile international normalized ratio (INR) (1 point); age > 65 years (1 point); and drugs (e.g., aspirin or non-steroidal anti-inflammatory drugs or alcohol) (1 point) [11].

The categorization of symptoms related to AF was carried out following the European Heart Rhythm Association’s AF symptom classification (EHRA score) [12] as follows: EHRA I, absence of symptoms; EHRA II, mild symptoms (no interference with normal daily activities); EHRA III, severe symptoms (interference with normal daily activities); EHRA IV, disabling symptoms (complete interference with daily activities). The EHRA score evaluates symptoms associated with AF, which diminish or disappear upon restoration of sinus rhythm or effective rate control and was determined by participating sites.

The evaluation of quality of life utilized the EuroQoL questionnaire, a validated tool that employs five aspects (mobility, self-care, typical activities, pain/discomfort, and anxiety/depression), each with five potential levels indicating the degree of issues (none, minimal, moderate, intense, and very intense). As noted earlier, patient responses at the outset were employed to derive a singular numerical value for each category, where higher values corresponded to poorer quality of life [13].

### 2.2. Rhythm Control Definitions

After the enrolment, all patients who received a rhythm control intervention such as electrical or pharmacological cardioversion, catheter ablation, or were prescribed an antiarrhythmic drug (Class Ia, Class Ic, Class III), were included in the ‘rhythm control’ group. All the other patients treated with Class II or Class IV antiarrhythmic drugs or digoxin were considered as on rate control strategies.

### 2.3. Study Outcomes

Adverse events were registered after 1-year of follow-up. The primary outcomes of the study were the risk of all-cause death, cardiovascular (CV) death, acute coronary syndrome, or significant coronary artery disease requiring percutaneous coronary intervention (ACS/PCI), new or worsening of a pre-existent heart failure, thromboembolic events, and major bleeding.

All-cause death was defined as death due to CV, non-CV, or unknown causes. CV death was defined as death due to fatal cardiac (ACS, heart failure, arrhythmia, cardiac perforation, tamponade, or other unspecified cardiac causes) or vascular (ischemic stroke, hemorrhagic stroke, systemic hemorrhages, peripheral embolism, and pulmonary embolism) events. Thromboembolic events were defined as the occurrence of stroke and/or systemic embolism. The occurrence of major bleeding was defined according to the ISTH definition [14].

### 2.4. Statistical Analyses

Continuous variables were reported as mean ± standard deviation. Categorical variables were reported as percentages. Normal distribution was assessed by the Kolmogorov-Smirnov test. Inter-group comparisons of continuous variables were made with Student’s *t* test while categorical variables were compared with χ^2^ test or Fisher exact test where needed. The incidence rate of adverse outcomes was calculated as the number of events/total person-years ratio and reported as incidence for 100 persons/year. Kaplan Meier curves with Log-rank test and Cox proportional hazard regression analyses were performed to investigate the association between DM and the 1-year risk of adverse outcomes in AF patients. The 1-year risks of adverse events were expressed as hazard ratios (HRs) with 95% CI. All the multivariable Cox regression analyses were adjusted for the variables considered into the CHA_2_DS_2_-VASc score (heart failure, hypertension, age, previous stroke, coronary or peripheral artery disease, and female sex) [10]. We performed 2 further sensitivity analyses, in the first, we added the following covariates to the main model: CKD, liver disease, and OAC use. In the second, we added CKD, liver disease, and vitamin K antagonists (VKA) use to the main model. 

Additionally, we performed an interaction analysis to assess the risk of all-cause death associated to DM in relevant subgroups (age < or ≥75 years, sex, paroxysmal AF, chronic kidney disease, OAC, and beta blockers). All the interaction analyses were adjusted for the same variables utilized in the main Cox-regression multivariable model. All tests were 2-tailed, and analyses were performed using computer software packages (SPSS-25.0, SPSS Inc., Chicago, IL, USA). A *p*-value < 0.05 was considered as statistically significant.

## 3. Results

**Figure 1:**
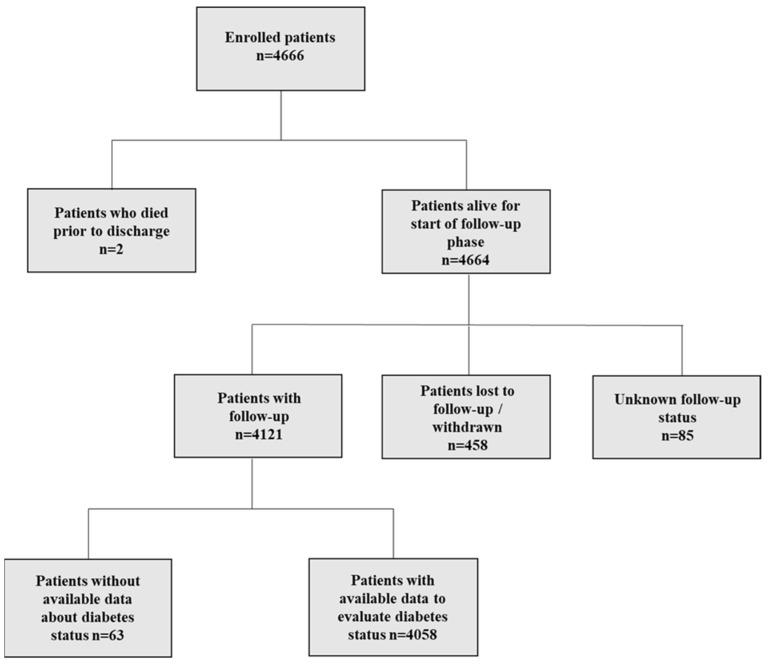
Patient flow of the study.

Amongst the 4666 patients enrolled in the APHRS registry, 4058 (86.9%) had available follow-up data and all the information related to their DM status (Figure 1). 

### 3.1. Clinical Characteristics

The final cohort comprised of 999 (24.6%) AF patients with DM (mean age 71 ± 11 years, 36.4% females, 99.6% with DM type 2) and 3059 (75.4%) AF patients without DM (mean age 68 ± 12 years, 33.7% females). Patients with DM were older, and with a higher prevalence of hypertension, dyslipidemia, heart failure, coronary and peripheral artery disease, CKD, liver disease, dementia, and previous hemorrhagic events compared to AF patients without DM (Table 1). 

The most frequent AF type was paroxysmal AF in patients without DM (43.7% vs. 37.2%), and permanent AF in those with DM (15.9% vs. 21.0%). DM was associated with a lower prevalence of severe and disabling AF related symptoms and with a worse quality of life, as shown by the higher mean value of 4 out of 5 EuroQoL domains (mobility, self-care, usual activities, and anxiety/depression) compared to patients without DM (Table 1). In AF patients with DM, 301 (30.1%) patients were not treated with any antidiabetic drugs, 596 (59.7%) were treated with oral antidiabetics, 59 (5.9%) with insulin and oral antidiabetics, and 43 (4.3%) with insulin only. 

### 3.2. Antithrombotic Management

AF patients with DM had a higher mean CHA_2_DS_2_-VASc and HAS-BLED score and were more often treated with OAC (85.5% vs. 81.5%, *p* = 0.003) antiplatelet drugs (18.5% vs. 14.0%, *p* < 0.001), and OAC with antiplatelet (9.7% vs. 5.1%, *p* < 0.001) than AF patients without DM. Among anticoagulated patients, those with DM had a significantly higher use of VKA compared to patients without DM (28.2% vs. 23.2%, *p* = 0.003) (Table 1).

The main reasons associated with non-use of oral anticoagulation therapy in AF patients with DM were patient being unwilling to take any OAC, previous bleeding, anemia, renal dysfunction, frequent falls, and recent hemorrhagic stroke, whilst in AF patients without DM was a low thromboembolic risk (see Table 1).

### 3.3. Rhythm Control

After the enrolment, in the whole population, 860 AF patients (21.2%) were treated with a rhythm control strategy and 3198 (79.8%) with rate control. Amongst patients in the rhythm control group, 187 (18.7%) AF patients with DM and 673 (22.0%) patients without DM were treated with rhythm control interventional procedures (*p* = 0.028). Patients with DM were less frequently treated with catheter ablation procedures (15.6% vs. 12.2%, *p* = 0.009) while no significant difference was found for the use of pharmacological (5.1% vs. 5.7%, *p* = 0.481) and electrical cardioversion (4.4% vs. 4.1%, *p* = 0.677) (Table 1).

### 3.4. Follow-Up

After 1-year of follow-up, the following events were recorded: 118 (2.9%) all-cause deaths, 26 (0.6%) cardiovascular deaths, 41 (1.0%) thromboembolic events, 27 (0.7%) new or 96 (2.4%) worsening HF events, and 46 (1.1%) major bleeding.

**Figure 2:**
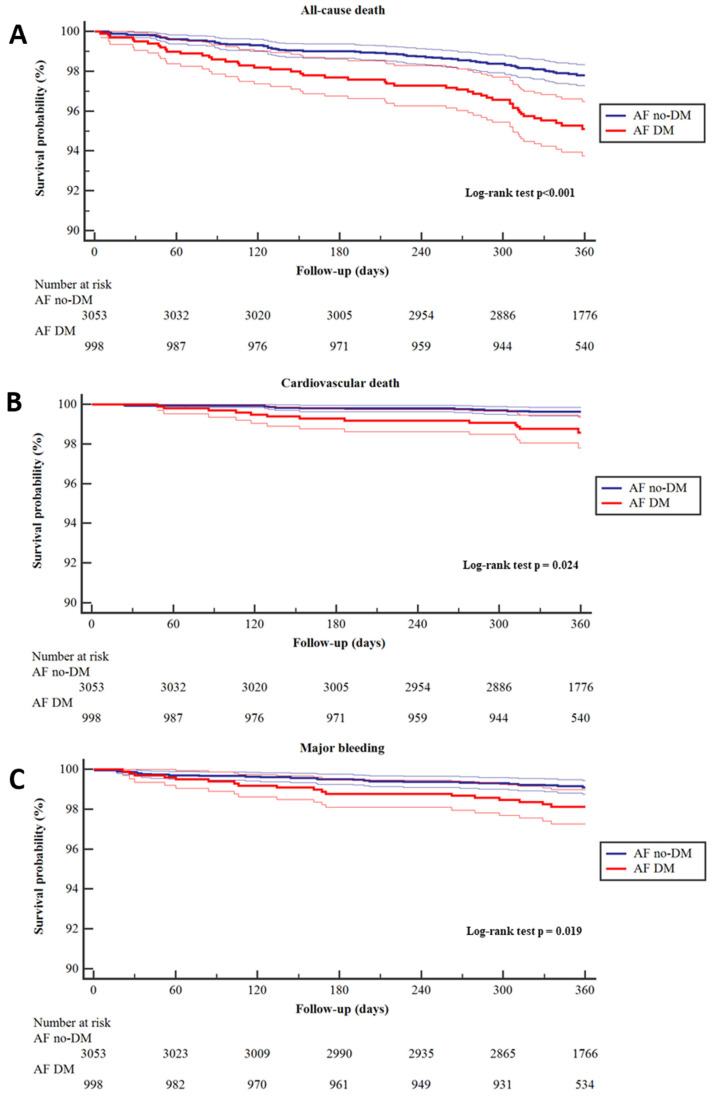
Kaplan-Meier curves for the risk of all-cause death (Panel (**A**)), cardiovascular death (Panel (**B**)), and major bleeding (Panel (**C**)) in patients with atrial fibrillation with and without diabetes. Legend: Thick lines (survival probability), thin lines (95% Confidene intervals). AF: Atrial Fibrillation, DM: Diabetes Mellitus.

Compared to AF patients without DM, those with DM had a higher annual incidence rate of all-cause death (2.3% vs. 5.0%, *p* < 0.001), cardiovascular death (0.4 vs. 1.3%, *p* = 0.023). and major bleeding (1.0% vs. 1.9%, *p* = 0.019) (Table 2). This higher risk was shown on univariable and multivariable Cox regression analysis (Table 2), where DM was associated with a higher risk of all-cause death (HR 1.48, 95% CI 1.00–2.19. Figure 2 Panel A), cardiovascular death (HR 2.33, 95% CI 1.01–5.40. Figure 2 Panel B), and major bleeding (HR 1.91, 95% 1.01–3.60. Figure 2 Panel C) after adjustment for heart failure, hypertension, age, previous stroke, coronary or peripheral artery disease, and female sex (Table 2, Supplementary Materials Tables S1, S2 and S6). No significant association between DM and the risk of ACS/PCI (Supplementary Materials Table S3), thromboembolic events (Supplementary Materials Table S4), new or worsening heart failure (Supplementary Materials Table S5) was found compared to AF patients without DM (Table 2).

### 3.5. Sensitivity Analysis

On Cox regression multivariable analysis built by adding to the main model the presence of liver disease, CKD, and OAC (Model 1. Table 2. Supplementary Materials Tables S7–S12) or VKA use (Model 2. Table 2. Supplementary Materials Tables S13–S18), we found that DM was associated with a significant higher risk of cardiovascular death (HR 2.38, 95% CI 1.02–5.52 and HR 2.61, 95% CI 1.05–6.51, for model 1 and model 2, respectively), and major bleeding (HR 1.90, 95% CI 1.01–3.58 and HR 2.08, 95% CI 1.09–3.99, for model 1 and model 2, respectively) in both models, whereas a non-significant trend was found for the risk of all-cause death (HR 1.33, 95% CI 0.88–2.00 and HR 1.40, 95% CI 0.87–2.26, for model 1 and model 2, respectively).

### 3.6. Subgroup Analyses

**Figure 3:**
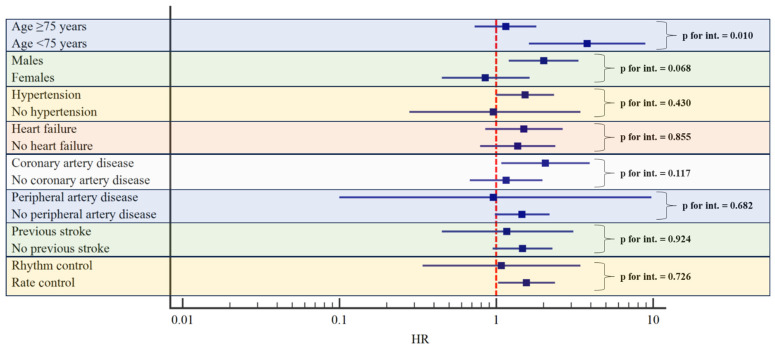
Risk of all-cause death according to the presence of diabetes in different subgroups. Adjusted for: age, sex, hypertension, heart failure, coronary artery disease, peripheral artery disease, and previous stroke. Legend: p for int.: *p*-value for interaction.

On subgroup analysis, we observed a statistically significant interaction between the age <75 years and the risk of all-cause death in patients with diabetes (HR 3.81, 95% CI 1.62–8.95 in patients <75 years vs. HR 1.15, 95% CI 0.73–1.80 in patients ≥75 years; *p* for interaction = 0.010). The effect of diabetes on the risk of mortality was also found higher in males compared to females, although without a statistically significant interaction (*p* for interaction = 0.068). No other statistically significant interactions were found for sex and the presence of hypertension, heart failure, coronary or peripheral artery disease, previous stroke, rate, or rhythm control approach (Figure 3, Supplementary Materials Table S19).

## 4. Discussion

The principal findings of our study are as follows: (1) The pooled prevalence of DM was 24.6%; (2) AF patients with DM had a high prevalence of comorbidities, worse quality of life (QOL), higher use of VKA, and lower use of rhythm control approaches; (3) the presence of DM was independently associated with higher risk of CV death and major bleeding; (4) the weight of DM in determining the risk of all-cause death in AF patients is more evident in younger than older patients.

The DM prevalence of about 25% we found in our Asian population was similar to the 23% showed in the EORP-AF registry conducted in European AF patients [15] and to the pooled prevalence of 26% reported in a recent metanalysis on more than 500,000 AF patients from different geographical areas [16], suggesting that ethnicity plays a marginal role on the differences for DM prevalence in AF patients. Moreover, also the clinical phenotype of AF patients with DM, characterized by advanced age, worse quality of life, and high prevalence of obesity and other cardiovascular risk factors, was consistent to those described in previous studies from European countries, underlying the clinical complexity of these patients, and explaining the higher use of OAC we found [17,18,19].

In our study there was a lower use of rhythm control approach among AF patients with DM comparing to those without DM. Although, in the last 20 years several studies investigated the “rhythm vs. rate” control strategy in AF patients failed to find apparent advantages in terms of mortality or stroke risk [20,21,22,23,24], more recent evidence suggests that an early rhythm control is associated with a significant reduction in the risk of CV events [25]. Thus, a larger use of this approach in patients with DM would be needed not only to clarify the potential benefit of this approach in this population, but also to evaluate the reliability of the patient-centered symptom-directed decisions in patients with diabetic neuropathies that can affect the typical symptomatic corollary. In patients with DM, the presence of left atrial cardiomyopathy is associated with increased cardiovascular morbidity and mortality [26]. Hence, counteracting these structural changes through early rhythm control strategies could ameliorate the short and long-term prognosis in these patients.

This body of evidence shows that patients with AF and DM need a careful and multifaceted approach to optimize their clinical management. The most recent European and APHRS guidelines for AF management [27,28] advocate the integrated ABC pathway, whereby the pillars of AF management include Avoiding stroke with Anticoagulation; Better management of the symptoms patient-centered symptom-directed decisions on rate or rhythm control; and Cardiovascular risk factor optimization and lifestyle changes. Adherence to the ABC pathway has been associated with improved outcomes, with lower risks of death, stroke, major bleeding and hospitalizations [29,30,31,32]; benefits of the ABC pathway were also observed in patients with AF and DM [33] including a post-hoc analysis of the mAFA-II cluster randomized trial, which shows similar benefit of a mobile health-implemented ABC pathway in reducing the risk of the primary outcome of death, stroke, thromboembolism and rehospitalization in patients with and without DM [34].

In our cohort, we found that DM was associated with a higher risk of all-cause death and CV death. This finding confirms the results of a metanalysis on more than 20 different studies that showed that DM in AF patients was associated with a 37% higher risk of all-cause death and a 46% higher risk of cardiovascular death compared to those without DM [16]; and of the Gulf-SAFE Registry study involving 2043 AF patients from the Middle East, in which DM was associated with a higher 1-year rates of all-cause death, heart failure and AF-related hospitalization than AF patients without DM [35]. However, our data seem to further suggest that Asian patients with DM have a greater risk of clinical complications and death compared to Western patients. Indeed, if the 48% increased risk of all-cause death we reported is similar to the pooled 43% reported in the above mentioned metanalysis, it is certainly higher than the 28% higher risk showed by AF patients with DM from the EORP-AF registry [15,16]. The same could be said for the 2-fold increased risk of cardiovascular death we have reported in our patients with DM, a value significantly higher than the 40% increased risk reported in studies performed on European AF populations [15,36]. Moreover, our results support other studies that have compared the risk of adverse events in Asians with DM with those from Europe. In a case control study on 292 DM patients, South Asians (56.5%) had a higher prevalence of macrovascular and microvascular complications and a higher risk of CV events compared to Europeans [37]. In a prospective cohort of 828 Asian patients and 27,962 non-Asians patients with insulin-treated DM followed for a mean of 28 years, the standardized mortality ratio in Asian patients was more than twice compared to non-Asians [38]. This was confirmed in another population-based sample of 730 south Asians and 304 Europeans followed by 11 years from the Southall Diabetes Survey, that reported a rate ratio (South Asian versus European) of 1.50 (95% CI 0.72–3.12) for all-cause mortality, 1.80 (95% CI 1.03–3.16) for CV death, and 2.02 (95% CI 1.04–3.92) for CV events [39].

In this study, DM beyond to the higher risk of all-cause death and CV death, was also associated with a higher risk of major bleeding. This relation was already reported by one postmarketing study on 44,793 rivaroxaban users with AF in which DM was associated with a higher annual rate of major bleeding compared to those without DM [40] and by a nationwide cohort study on 326,832 Swedish AF patients, where DM was associated with a higher risk of death, cardiovascular events, and major bleeding (HR 1.12, 95% CI 1.06–1.19) [41]. In our patients, the higher risk of bleeding in patients with DM could be explained by the significantly higher baseline median HAS-BLED score, the greater OAC use (especially VKA) and the higher CKD prevalence. However, when we add to the main multivariable models the OAC use or the OAC type (VKA or NOAC), and CKD, DM was still associated with a significantly high-risk of major bleeding showing the robustness of our results. This underlines the need for personalized antithrombotic approaches aimed at reducing the risk of hemorrhages in AF patients with DM. Indeed, the VKA use in these patients should be limited for the possible CKD progression due to vascular calcification [42], and the timing for combined antithrombotic therapies with OAC and antiplatelets in patients with DM, AF, and coronary disease should be carefully evaluated to achieve the best net clinical benefit between the risk of recurrent thrombosis and that for major bleeding [27].

The reasons behind the higher risk of adverse events in Asians AF patients compared to Europeans are largely unknown but probably related to the interaction between genetic, environmental, and socio-economic factors [43,44]. However, a possible other explanation to this discrepancy could be given by the 10 years younger age at DM diagnosis reported in Asians compared to Europeans (42 ± 11.5 years vs. 52.9 ± 12) [45]. Indeed, the early DM onset in Asians may lead to an anticipate occurrence of macro and microvascular complication that worsen the clinical course of these young patients even more than what occurs in older where the DM has raised later. This hypothesis seems to be confirmed by the interaction analysis where the impact of DM in determining the risk of all-cause death was significantly greater in patients with <75 years compared to those with ≥75 years. Indeed, older patients have a high risk of death per se, resulting from the coexistence of several physiological and pathological conditions and therefore the relative contribution of DM is expected to be lower. On the other hand, in younger patients, DM has a more powerful driver of prognosis, since these individuals have a lower baseline risk of death, and the presence of each CV risk factor increases considerably the overall risk of adverse events.

This study is limited by its observational design; DM status was evaluated only at enrollment, and not during follow-up, therefore possible new diagnoses of DM could be overlooked. Moreover, no routine oral glucose tolerance testing and HbA1c was evaluated at the time of enrollment so we could have missed some cases of DM. No data are available about the most recent antidiabetic treatments with sodium–glucose cotransporter-2 (SGLT2) inhibitor or a glucagon-like peptide-1 (GLP-1) receptor agonist because the study enrolment period was before the publication of the most recent guidelines for DM management [46]. We did not have information on quality of INR control which is reflected by therapeutic range for patients treated with VKA what could be important since patients with DM were more commonly treated with this type of OAC. Furthermore, we adjusted mortality, major bleeding, ACS/PCI, and thromboembolic events analyses by only using CHA_2_DS_2_-VASc score parameters extended by the use of OAC or OAC type (VKA vs. NOAC) and CKD; thus, residual confounding may still exist. Finally, when interpreting the results of this study, we must consider that the low incidence of cardiovascular events, the relatively small sample size, the short follow-up, and the unequal sample sizes can result in reduced statistical power.

Despite all these limitations, this study reports important data about the clinical management and the risk of adverse events in a large multinational cohort of Asian patients with AF and thus have a crucial impact in balancing the Asians underrepresentation into the studies on high-impact cardiometabolic conditions performed over the past 10 years [47].

In conclusion, given the high rates of cardiovascular events and major bleeding in these Asian AF patients with DM, efforts to optimize the management approach of these high-risk patients in a holistic or integrated care approach that considers the presence of ethnic-specific characteristics are needed.

## Figures and Tables

**Table 1:** Baseline Characteristics and Medication Use Stratified by Diabetes Mellitus.

	No Diabetes (*n* = 3059)	Diabetes (*n* = 999)	*p*-Value
Age (years), mean ± SD	67.6 ± 12.1	71.4 ± 10.6	<0.001
Women, *n* (%)	1031 (33.7)	364 (36.4)	0.114
**Medical examination, mean ± SD**			
Heart rate (bpm)	76.3 ± 16.3	77.5 ± 16.3	0.057
Systolic blood pressure (mmHg)	127.8 ± 18.4	132.6 ± 18.7	<0.001
Dyastolic blood pressure (mmHg)	74.7 ± 12.3	73.6 ± 12.5	0.010
BMI (kg/m^2^)	24.6 ± 4.04	26.2 ± 4.5	<0.001
**Comorbidities, *n* (%)**			
Hypertension	1684 (55.4)	795 (79.9)	<0.001
Dyslipidaemia	985 (32.5)	560 (56.7)	<0.001
Heart Failure	593 (19.6)	255 (26.0)	<0.001
Coronary artery disease	499 (16.6)	282 (28.8)	<0.001
Ischaemic stroke/transient ischemic attack	285 (9.4)	106 (10.7)	0.223
Haemorrhagic stroke	44 (1.4)	26 (2.6)	0.014
Haemorrhagic event	213 (7.0)	92 (9.3)	0.019
Chronic Kidney disease	173 (5.7)	135 (13.5)	<0.001
Chronic obstructive pulmonary disease	84 (2.8)	28 (2.8)	0.924
Sleep apnoea	94 (3.1)	36 (3.6)	0.408
Peripheral artery disease	31 (1.0)	21 (2.1)	0.008
Cancer	66 (2.2)	29 (2.9)	0.176
Liver disease	113 (3.7)	70 (7.0)	<0.001
Dementia	40 (1.3)	32 (3.2)	<0.001
Current smoking	245 (8.0)	106 (10.6)	0.011
**Medications, *n* (%)**			
ACE inhibitor	348 (11.4)	188 (18.9)	<0.001
Aldosterone blocker	192 (6.3)	73 (7.3)	0.507
ARB	727 (23.9)	337 (33.8)	<0.001
Beta-blocker	1482 (48.7)	565 (56.8)	<0.001
CCB	699 (22.9)	245 (24.6)	0.281
CCB-non DHP	413 (13.5)	146 (14.6)	0.658
Digoxin	333 (10.9)	121 (12.1)	0.284
Diuretic	675 (22.1)	219 (22.0)	0.936
Statin	983 (32.3)	550 (55.4)	<0.001
**AF classification, *n* (%)**			
First detected	208 (6.8)	84 (8.4)	
Paroxysmal	1333 (43.7)	371 (37.2)	
Persistent	745 (24.4)	229 (23.0)	<0.001
Long-standing persistent	279 (9.1)	103 (10.3)	
Permanent	485 (15.9)	209 (21.0)	
**EHRA classification, *n* (%)**			
EHRA I (no symptoms)	1885 (61.6)	712 (71.3)	
EHRA II (mild symptoms)	962 (31.4)	239 (23.9)	
EHRA III (severe symptoms)	186 (6.1)	45 (4.5)	<0.001
EHRA IV (disabling symptoms)	26 (0.8)	3 (0.3)	
**Symptoms, *n* (%)**			
Palpitations	765 (25.0)	159 (15.9)	<0.001
Dyspnoea	343 (11.2)	92 (9.2)	0.076
Fatigue	119 (3.9)	42 (4.2)	0.659
Non-wellbeing	58 (1.9)	14 (1.4)	0.304
Dizziness	225 (7.4)	66 (6.6)	0.426
Syncope	55 (1.8)	7 (0.7)	0.014
Chest pain	180 (5.9)	62 (6.2)	0.709
Fear/anxiety	60 (2.0)	9 (0.9)	0.024
**EuroQoL, mean ± SD**			
Mobility	1.30 ± 0.67	1.45 ± 0.84	<0.001
Self-care	1.14 ± 0.52	1.24 ± 0.72	<0.001
Usual Activities	1.24 ± 0.61	1.33 ± 0.80	<0.002
Pain/Discomfort	1.41 ± 0.67	1.44 ± 0.68	0.319
Anxiety/Depression	1.38 ± 0.68	1.32 ± 0.61	0.018
**Thrombotic and hemorrhagic risk**			
CHA_2_DS_2_-VASc, mean ± SD	2.3 ± 1.6	4 ± 1.5	<0.001
CHA_2_DS_2_-VASc ≥ 2, *n* (%)	2000 (65.4)	968 (96.9)	<0.001
HAS-BLED, mean ± SD	1.3 ± 1.0	1.7 ± 1.1	<0.001
HAS-BLED ≥ 3, *n* (%)	353 (11.5)	206 (20.6)	<0.001
**Antithrombotic treatment, *n* (%)**			
Any antiplatelet	427 (14.0)	185 (18.5)	<0.001
Any anticoagulant	2494 (81.5)	855 (85.5)	0.003
Any anticoagulant–any antiplatelet	157 (5.1)	97 (9.7)	<0.001
Vitamin K antagonist	578 (23.2)	241 (28.2)	0.003
NOACs	1916 (76.8)	614 (71.8)	
Apixaban	558 (18.2)	197 (19.7)	0.297
Dabigatran	354 (11.4)	132 (13.2)	0.166
Edoxaban	326 (10.7)	69 (6.9)	0.001
Rivaroxaban	678 (22.2)	216 (21.6)	0.719
**Reasons for not using any OAC**			
No indication (low risk), *n* (%)	276 (48.8)	40 (27.8)	<0.001
Unwilling to take any OAC, *n* (%)	82 (14.5)	32 (22.2)	0.025
Prior bleeding, *n* (%)	20 (3.5)	11 (7.6)	0.032
OAC not considered adequate by physician despite stroke risk, *n* (%)	5 (0.9)	5 (3.5)	0.190
Recent/planned surgery/intervention, *n* (%)	19 (3.4)	9 (6.3)	0.112
Active peptic ulcer, *n* (%)	4 (0.7)	1 (0.7)	0.986
Anemia, *n* (%)	22 (3.9)	14 (9.7)	0.004
Thrombocytopenia, *n* (%)	4 (0.7)	0 (0.0)	0.311
Renal dysfunction, *n* (%)	12 (2.1)	13 (9.0)	<0.001
Liver disease, *n* (%)	2 (0.4)	1 (0.7)	0.574
Malignancy, *n* (%)	8 (1.4)	2 (1.4)	0.980
Alcohol or drug abuse or psychosocial issues, *n* (%)	2 (0.4)	0 (0.0)	0.475
Frequent falls, *n* (%)	6 (1.1)	5 (3.5)	0.037
Dementia, *n* (%)	2 (0.4)	0 (0.0)	0.475
Recent hemorrhagic stroke, *n* (%)	1 (0.2)	3 (2.1)	0.006
Intolerance/allergy, *n* (%)	3 (0.5)	0 (0.0)	0.381
Other, *n* (%)	63 (11.2)	16 (11.1)	0.989
**Rhythm control strategies**			
Antiarrhythmics, *n* (%)	701 (23.1)	215 (21.6)	0.319
Amiodarone, *n* (%)	238 (7.8)	79 (7.9)	0.904
Dronedarone, *n* (%)	71 (2.3)	29 (2.9)	0.304
Flecainide, *n* (%)	146 (4.8)	33 (3.3)	0.049
Propafenone, *n* (%)	215 (7.0)	72 (7.2)	0.854
Sotalol, *n* (%)	56 (1.8)	15 (1.5)	0.489
Disopyramide, *n* (%)	4 (0.1)	2 (0.2)	0.124
Quinidine, *n* (%)	1 (0.0)	0 (0.0)	0.567
Interventional procedures, *n* (%)	673 (22.0)	187 (18.7)	0.028
Electrical cardioversion, *n* (%)	135 (4.4)	41 (4.1)	0.677
Pharmacological cardioversion, *n* (%)	157 (5.1)	57 (5.7)	0.481
Catheter ablation, *n* (%)	477 (15.6)	122 (12.2)	0.009

Legend: ACE i: Angiotensin receptor inhibitor; ARB: Angiotensin receptor blocker; BMI: Body Mass Index; bpm: beats per minute; CCB: calcium channel blocker; CCB-non DHP: calcium channel blocker- non dihidropiridine; OAC: oral anticoagulant; NOAC: non-vitamin K oral anticoagulant.

**Table 2:** Adverse events at 1-year follow-up in relation to the presence of diabetes.

	No Diabetes (*n* = 3059) Number of Events (Incidence/100 Persons/Year)	Diabetes (*n* = 999) Number of Events (Incidence/100 Persons/Year)	*p*-Value	Univariable Analysis HR (95% CI)	Multivariable Analysis * HR (95% CI)	Sensitivity Analysis Multivariable Model 1 HR (95% CI)	Sensitivity Analysis Multivariable Model 2 HR (95% CI)
All–cause death	69 (2.3)	49 (5.0)	<0.001	2.02 (1.53–3.18)	1.48 (1.00–2.19)	1.33 (0.88–2.00)	1.40 (0.87–2.26)
CV death	13 (0.4)	13 (1.3)	0.023	3.10 (1.44–6.69)	2.33 (1.01–5.40)	2.38 (1.02–5.52)	2.61 (1.05–6.51)
ACS/PCI	29 (1.0)	12 (1.2)	0.465	1.31 (0.67–2.56)	0.95 (0.47–1.92)	0.99 (0.48–2.01)	0.73 (0.30–1.77)
Thromboembolic event	19 (0.6)	8 (0.8)	0.521	1.39 (0.61–3.19)	1.42 (0.60–3.37)	1.43 (0.59–3.44)	1.36 (0.50–3.73)
New or worsening HF	68 (2.3)	28 (2.9)	0.275	1.28 (0.83–1.99)	0.87 (0.53–1.41)	0.87 (0.53–1.41)	0.82 (0.47–1.42)
Major bleeding	28 (1.0)	18 (1.9)	0.019	1.99 (1.10–3.60)	1.91 (1.01–3.60)	1.90 (1.01–3.58)	2.08 (1.09–3.99)

* Adjusted for age, sex, heart failure, hypertension, coronary and peripheral artery diseases, and previous thromboembolic events. **Model 1:** adjusted for the same variables of the main model and CKD, liver disease and OAC. **Model 2:** adjusted for the same variables of the main model and CKD, liver disease and VKA. Legend: ACS/PCI: Acute Coronary Syndrome or significant coronary artery disease requiring Percutaneous Coronary Intervention, CKD: Chronic Kidney Disease, CV: Cardio-Vascular, HF: Heart Failure, OAC: Oral anticoagulant, VKA: Vitamin-K antagonist.

## Data Availability

The data underlying this article will be shared on reasonable request to the corresponding author.

## Supplementary Materials

The following supporting information can be downloaded at: https://www.mdpi.com/article/10.3390/jcm13051274/s1, Table S1. Cox regression multivariable analysis for the risk of all-cause of death in patients with atrial fibrillation and diabetes; Table S2. Cox regression multivariable analysis for the risk of cardiovascular death in patients with atrial fibrillation and diabetes; Table S3. Cox regression multivariable analysis for the risk of acute coronary syndrome or percutaneous coronary interventions in patients with atrial fibrillation and diabetes; Table S4. Cox regression multivariable analysis for the risk of thromboembolic events in patients with atrial fibrillation and diabetes; Table S5. Cox regression multivariable analysis for the risk of new or worsening heart failure in patients with atrial fibrillation and diabetes; Table S6. Cox regression multivariable analysis for the risk of major bleeding in patients with atrial fibrillation and diabetes; Table S7. Cox regression multivariable sensitivity analysis for the risk of all-cause death in patients with atrial fibrillation and diabetes (Model 1); Table S8. Cox regression multivariable sensitivity analysis for the risk of cardiovascular death in patients with atrial fibrillation and diabetes (Model 1); Table S9. Cox regression multivariable sensitivity analysis for the risk of acute coronary syndrome or percutaneous coronary interventions in patients with atrial fibrillation and diabetes (Model 1); Table S10. Cox regression multivariable sensitivity analysis for the risk of Thromboembolic event in patients with atrial fibrillation and diabetes (Model 1); Table S11. Cox regression multivariable sensitivity analysis for the risk of new or worsening heart failure in patients with atrial fibrillation and diabetes (Model 1); Table S12. Cox regression multivariable sensitivity analysis for the risk of major bleeding in patients with atrial fibrillation and diabetes (Model 1); Table S13. Cox regression multivariable sensitivity analysis for the risk of all-cause death in patients with atrial fibrillation and diabetes (Model 2); Table S14. Cox regression multivariable sensitivity analysis for the risk of cardiovascular death in patients with atrial fibrillation and diabetes (Model 2); Table S15. Cox regression multivariable sensitivity analysis for the risk of acute coronary syndrome or percutaneous coronary interventions in patients with atrial fibrillation and diabetes (Model 2); Table S16. Cox regression multivariable sensitivity analysis for the risk of thromboembolic events in patients with atrial fibrillation and diabetes (Model 2); Table S17. Cox regression multivariable sensitivity analysis for the risk of new or worsening heart failure in patients with atrial fibrillation and diabetes (Model 2); Table S18. Cox regression multivariable sensitivity analysis for the risk of major bleeding in patients with atrial fibrillation and diabetes (Model 2); Table S19. Interaction analysis for the risk of all-cause death, cardiovascular death, and major bleeding in patients with atrial fibrillation and diabetes mellitus.
