# Supplementary material for: Adverse Events and Clinical Correlates in Asian Patients with Atrial Fibrillation and Diabetes Mellitus: A Report from Asia Pacific Heart Rhythm Society Atrial Fibrillation Registry
Source: J Clin Med. 2024 Feb 23;13(5):1274. doi: 10.3390/jcm13051274 (PMC10932296; doi:10.3390/jcm13051274)
Supplement: Supplementary file 1 [file jcm-13-01274-s001.zip › jcm-2792286-supplementary.pdf]

Adverse events and clinical corelates in Asian patients with atrial fibrillation and type 2 diabetes mellitus: A report from Asia Pacific Heart Rhythm Society Atrial Fibrillation Registry.

Tommaso Bucci, Katarzyna Nabrdalik, Alena Shantsila, Giulio Francesco Romiti, Wee-Siong Teo, Hyung-Wook Park, Wataru Shimizu, Hung-Fat Tse, Marco Proietti, Tze-Fan Chao, Gregory Y. H. Lip; Asia-Pacific Heart Rhythm Society Atrial Fibrillation Registry Investigators[§].

Supplementary materials

Supplementary Table 1. Cox regression multivariable analysis for the risk of all-cause of death in patients with atrial fibrillation and diabetes.

	**HR**	**95%CI**	**p-value**
Age	1.09	1.07-1.11	<0.001
Female sex	0.99	0.67-1.44	0.908
Hypertension	1.52	0.92-2.51	0.101
Diabetes	1.48	1.00-2.19	0.050
Heart Failure	2.75	1.88-4.01	<0.001
Coronary artery disease	1.67	1.12-2.48	0.011
Peripheral artery disease	2.50	1.01-6.20	0.048
Previous stroke	1.43	0.86-2.37	0.167

HR: Hazard Ratio, CI: Confidence Interval.

Supplementary Table 2. Cox regression multivariable analysis for the risk of cardiovascular death in patients with atrial fibrillation and diabetes.

	**HR**	**95%CI**	**p-value**
Age	1.08	(1.03-1.12)	0.001
Female sex	0.97	(0.42-2.23)	0.937
Hypertension	1.14	(0.41-3.17)	0.805
Heart Failure	3.77	(1.65-8.61)	0.002
Diabetes	2.33	(1.01-5.40)	0.047
Coronary artery disease	2.52	(1.10-5.75)	0.029
Peripheral artery disease	1.87	(0.25-14.14)	0.543
Previous stroke	1.04	(0.31-3.48)	0.954

HR: Hazard Ratio, CI: Confidence Interval.

Supplementary Table 3. Cox regression multivariable analysis for the risk of acute coronary syndrome or percutaneous coronary interventions in patients with atrial fibrillation and diabetes.

	**HR**	**95%CI**	**p-value**
Age	1.03	(1.00-1.06)	0.091
Female sex	0.81	(0.40-1.62)	0.543
Hypertension	2.39	(1.03-5.57)	0.043
Heart Failure	1.02	(0.49-2.12)	0.951
Diabetes	0.95	(0.47-1.92)	0.895
Coronary artery disease	2.76	(1.43-5.29)	0.002
Peripheral artery disease	1.47	(0.20-10.9)	0.704
Previous stroke	0.41	(0.10-1.71)	0.221

HR: Hazard Ratio, CI: Confidence Interval.

Supplementary Table 4. Cox regression multivariable analysis for the risk of thromboembolic events in patients with atrial fibrillation and diabetes.

	**HR**	**95%CI**	**p-value**
Age	1.04	(1.00-1.08)	0.031
Female sex	1.49	(0.68-3.27)	0.322
Hypertension	0.86	(0.37-1.98)	0.715
Heart Failure	2.48	(1.11-5.56)	0.028
Diabetes	1.42	(0.60-3.37)	0.426
Coronary artery disease	0.30	(0.07-1.25)	0.096
Peripheral artery disease	-	-	-
Previous stroke	0.75	(0.18-3.21)	0.701

HR: Hazard Ratio, CI: Confidence Interval.

Supplementary Table 5. Cox regression multivariable analysis for the risk of new or worsening heart failure in patients with atrial fibrillation and diabetes.

	**HR**	**95%CI**	**p-value**
Age	1.02	(1.00-1.04)	0.053
Female sex	1.40	(0.90-2.18)	0.136
Hypertension	1.46	(0.86-2.46)	0.161
Heart Failure	2.98	(1.94-4.60)	<0.001
Diabetes	0.90	(0.56-1.45)	0.657
Coronary artery disease	2.59	(1.66-4.05)	<0.001
Peripheral artery disease	3.19	(1.16-8.80)	0.025
Previous stroke	1.37	(0.76-2.49)	0.297

HR: Hazard Ratio, CI: Confidence Interval.

Supplementary Table 6. Cox regression multivariable analysis for the risk of major bleeding in patients with atrial fibrillation and diabetes.

	**HR**	**95%CI**	**p-value**
Age	1.06	(1.03-1.10)	<0.001
Female sex	1.07	(0.58-2.00)	0.828
Hypertension	1.16	(0.57-2.39)	0.679
Heart Failure	1.17	(0.58-2.35)	0.661
Diabetes	1.91	(1.01-3.60)	0.046
Coronary artery disease	0.61	(0.27-1.39)	0.238
Peripheral artery disease	3.33	(0.79-13.94)	0.100
Previous stroke	1.31	(0.55-3.14)	0.537

HR: Hazard Ratio, CI: Confidence Interval.

Supplementary Table 7. Cox regression multivariable sensitivity analysis for the risk of all-cause death in patients with atrial fibrillation and diabetes (Model 1).

	**HR**	**95%CI**	**p-value**
Age	1.09	(1.06-1.11)	<0.001
Female sex	0.92	(0.62-1.36)	0.663
Hypertension	1.48	(0.89-2.46)	0.130
Heart Failure	2.53	(1.71-3.73)	<0.001
Diabetes	1.33	(0.88-2.00)	0.171
Coronary artery disease	1.48	(0.99-2.23)	0.057
Peripheral artery disease	1.78	(0.70-4.55)	0.225
Previous stroke	1.60	(0.96-2.67)	0.071
Liver disease	0.84	(0.34-2.09)	0.710
Chronic kidney disease	1.77	(1.09-2.87)	0.020
Oral anticoagulant	0.46	(0.31-0.73)	0.001

HR: Hazard Ratio, CI: Confidence Interval.

Supplementary Table 8. Cox regression multivariable sensitivity analysis for the risk of cardiovascular death in patients with atrial fibrillation and diabetes (Model 1).

	**HR**	**95%CI**	**p-value**
Age	1.07	(1.03-1.12)	0.001
Female sex	1.00	(0.43-1.12)	1.000
Hypertension	1.13	(0.41-3.16)	0.811
Heart Failure	3.72	(1.62-8.57)	0.002
Diabetes	2.38	(1.02-5.52)	0.044
Coronary artery disease	2.50	(1.09-5.73)	0.031
Peripheral artery disease	1.62	(0.20-13.70)	0.650
Previous stroke	1.02	(0.30-3.44)	0.977
Liver disease	-	-	-
Chronic kidney disease	1.31	(0.44-3.89)	0.626
Oral anticoagulant	1.22	(0.39-3.76)	0.732

HR: Hazard Ratio, CI: Confidence Interval.

Supplementary Table 9. Cox regression multivariable sensitivity analysis for the risk of acute coronary syndrome or percutaneous coronary interventions in patients with atrial fibrillation and diabetes (Model 1).

	**HR**	**95%CI**	**p-value**
Age	1.03	(1.00-1.06)	0.107
Female sex	0.83	(0.41-1.68)	0.611
Hypertension	2.39	(1.02-5.62	0.046
Heart Failure	0.91	(0.42-1.95)	0.798
Diabetes	0.99	(0.48-2.01)	0.955
Coronary artery disease	2.58	(1.33-5.03)	0.005
Peripheral artery disease	1.41	(0.19-10.66)	0.742
Previous stroke	0.46	(0.11-1.92)	0.286
Liver disease	1.22	(0.29-5.15)	0.784
Chronic kidney disease	1.26	(0.46-3.41)	0.656
Oral anticoagulant	0.49	(0.24-0.99)	0.047

HR: Hazard Ratio, CI: Confidence Interval.

Supplementary Table 10. Cox regression multivariable sensitivity analysis for the risk of Thromboembolic event in patients with atrial fibrillation and diabetes (Model 1).

	**HR**	**95%CI**	**p-value**
Age	1.04	(1.01-1.08)	0.027
Female sex	1.51	(0.68-3.31)	0.309
Hypertension	0.85	(0.37-1.98)	0.706
Heart Failure	2.52	(1.11-5.72)	0.026
Diabetes	1.43	(0.59-3.44)	0.429
Coronary artery disease	0.28	(0.06-1.19)	0.084
Peripheral artery disease	-	-	-
Previous stroke	0.84	(0.20-3.63)	0.753
Liver disease	0.72	(0.10-5.45)	0.753
Chronic kidney disease	1.13	(0.32-3.97)	0.851
Oral anticoagulant	0.46	(0.19-1.12)	0.088

HR: Hazard Ratio, CI: Confidence Interval.

Supplementary Table 11. Cox regression multivariable sensitivity analysis for the risk of new or worsening heart failure in patients with atrial fibrillation and diabetes (Model 1).

	**HR**	**95%CI**	**p-value**
Age	1.02	(1.00-1.04)	0.093
Female sex	1.35	(0.86-2.11)	0.192
Hypertension	1.41	(0.83-2.39)	0.208
Heart Failure	2.91	(1.87-4.54)	<0.001
Diabetes	0.87	(0.53-1.41)	0.560
Coronary artery disease	2.43	(1.54-3.82)	<0.001
Peripheral artery disease	2.56	(0.90-7.28)	0.077
Previous stroke	1.29	(0.69-2.39)	0.426
Liver disease	0.68	(0.21-2.17)	0.513
Chronic kidney disease	1.82	(1.02-3.26)	0.044
Oral anticoagulant	0.84	(0.49-1.47)	0.548

HR: Hazard Ratio, CI: Confidence Interval.

Supplementary Table 12. Cox regression multivariable sensitivity analysis for the risk of major bleeding in patients with atrial fibrillation and diabetes (Model 1).

	**HR**	**95%CI**	**p-value**
Age	1.07	(1.03-1.10)	<0.001
Female sex	1.08	(0.58-2.01)	0.814
Hypertension	1.17	(0.57-2.40)	0.665
Heart Failure	1.21	(0.60-2.43)	0.592
Diabetes	1.90	(1.01-3.58)	0.047
Coronary artery disease	0.66	(0.29-1.51)	0.327
Peripheral artery disease	3.86	(0.91-16.33)	0.066
Previous stroke	1.19	(0.50-2.85)	0.693
Liver disease	2.11	(0.74-5.98)	0.162
Chronic kidney disease	0.57	(0.17-1.92)	0.363
Oral anticoagulant	2.31	(0.71-7.54)	0.165

HR: Hazard Ratio, CI: Confidence Interval.

Supplementary Table 13. Cox regression multivariable sensitivity analysis for the risk of all-cause death in patients with atrial fibrillation and diabetes (Model 2).

	**HR**	**95%CI**	**p-value**
Age	1.09	(1.06-1.12)	<0.001
Female sex	0.91	(0.57-1.45)	0.681
Hypertension	1.36	(0.75-2.49)	0.316
Heart Failure	2.19	(1.37-3.48)	0.001
Diabetes	1.40	(0.87-2.26)	0.164
Coronary artery disease	1.40	(0.86-2.30)	0.178
Peripheral artery disease	2.17	(0.66-7.12)	0.200
Previous stroke	1.33	(0.74-2.40)	0.338
Liver disease	0.69	(0.21-2.21)	0.527
Chronic kidney disease	1.49	(0.81-2.74)	0.205
Vitamin-K antagonist	3.83	(2.39-6.13)	<0.001

HR: Hazard Ratio, CI: Confidence Interval.

Supplementary Table 14. Cox regression multivariable sensitivity analysis for the risk of cardiovascular death in patients with atrial fibrillation and diabetes (Model 2).

	**HR**	**95%CI**	**p-value**
Age	1.08	(1.03-1.13)	0.002
Female sex	1.06	(0.43-2.63)	0.905
Hypertension	0.83	(0.29-2.40)	0.728
Heart Failure	3.11	(1.27-7.66)	0.013
Diabetes	2.61	(1.05-6.51)	0.039
Coronary artery disease	2.12	(0.85-5.27)	0.108
Peripheral artery disease	2.42	(0.30-19.47)	0.406
Previous stroke	1.08	(0.31-3.73)	0.905
Liver disease	-	-	-
Chronic kidney disease	1.54	(0.48-4.93)	0.466
Vitamin-K antagonist	2.43	(0.99-5.98)	0.053

HR: Hazard Ratio, CI: Confidence Interval.

Supplementary Table 15. Cox regression multivariable sensitivity analysis for the risk of acute coronary syndrome or percutaneous coronary interventions in patients with atrial fibrillation and diabetes (Model 2).

	**HR**	**95%CI**	**p-value**
Age	1.02	(0.99-1.06)	0.236
Female sex	0.52	(0.21-1.30)	0.162
Hypertension	3.04	(1.02-9.02)	0.045
Heart Failure	0.65	(0.24-1.74)	0.389
Diabetes	0.73	(0.30-1.77)	0.483
Coronary artery disease	2.09	(0.93-4.66)	0.074
Peripheral artery disease	2.57	(0.34-19.6)	0.363
Previous stroke	0.54	(0.13-2.30)	0.405
Liver disease	1.68	(0.40-7.17)	0.481
Chronic kidney disease	0.74	(0.17-3.26)	0.692
Vitamin-K antagonist	1.79	(0.81-3.99)	0.153

HR: Hazard Ratio, CI: Confidence Interval.

Supplementary Table 16. Cox regression multivariable sensitivity analysis for the risk of thromboembolic events in patients with atrial fibrillation and diabetes (Model 2).

	**HR**	**95%CI**	**p-value**
Age	1.04	(1.00-1.09)	0.076
Female sex	2.00	(0.79-5.06)	0.143
Hypertension	0.87	(0.33-2.32)	0.779
Heart Failure	2.15	(0.82-5.63)	0.120
Diabetes	1.36	(0.50-3.73)	0.545
Coronary artery disease	0.44	0.10-1.95)	0.278
Peripheral artery disease	-	-	-
Previous stroke	0.93	(0.21-4.07)	0.918
Liver disease	0.97	(0.13-7.52)	0.978
Chronic kidney disease	1.29	(0.28-5.83)	0.743
Vitamin-K antagonist	1.17	(0.41-3.36)	0.768

HR: Hazard Ratio, CI: Confidence Interval.

Supplementary Table 17. Cox regression multivariable sensitivity analysis for the risk of new or worsening heart failure in patients with atrial fibrillation and diabetes (Model 2).

	**HR**	**95%CI**	**p-value**
Age	1.02	(1.00-1.04)	0.121
Female sex	1.05	(0.64-1.75)	0.838
Hypertension	1.24	(0.70-2.17)	0.463
Heart Failure	2.36	(1.44-3.86)	0.001
Diabetes	0.82	(0.47-1.42)	0.469
Coronary artery disease	2.25	(1.36-3.74)	0.002
Peripheral artery disease	0.82	(0.11-5.85)	0.818
Previous stroke	1.37	(0.71-2.63)	0.343
Liver disease	0.51	(0.12-2.09)	0.349
Chronic kidney disease	1.57	(0.78-3.16)	0.203
Vitamin-K antagonist	1.80	(1.10-2.95)	0.020

HR: Hazard Ratio, CI: Confidence Interval.

Supplementary Table 18. Cox regression multivariable sensitivity analysis for the risk of major bleeding in patients with atrial fibrillation and diabetes (Model 2).

	**HR**	**95%CI**	**p-value**
Age	1.07	(1.03-1.10)	<0.001
Female sex	1.10	(0.58-2.09)	0.777
Hypertension	1.30	(0.60-2.82)	0.502
Heart Failure	0.96	(0.45-2.05)	0.917
Diabetes	2.08	(1.09-3.99)	0.027
Coronary artery disease	0.73	(0.32-1.68)	0.456
Peripheral artery disease	4.18	(0.98-17.3)	0.053
Previous stroke	1.15	(0.48-2.78)	0.755
Liver disease	2.20	(0.77-6.26)	0.141
Chronic kidney disease	0.40	(0.09-1.71)	0.215
Vitamin-K antagonist	1.86	(0.97-3.59)	0.064

HR: Hazard Ratio, CI: Confidence Interval.

Supplementary Table 19. Interaction analysis for the risk of all-cause death, cardiovascular death, and major bleeding in patients with atrial fibrillation and diabetes mellitus.

		All-cause death	
		HR 95%CI	p for interaction
Age≥75	DM	1.15 (0.73-1.80)	0.010
	No DM	Reference	
Age<75	DM	3.81 (1.62-8.95)	
	No DM	Reference	
Males	DM	2.01 (1.20-3.35)	0.068
	No DM	Reference	
Females	DM	0.85 (0.45-1.63)	
	No DM	Reference	
Hypertension	DM	1.53 (1.00-2.34)	0.430
	No DM	Reference	
No hypertension	DM	0.96 (0.28-3.45)	
	No DM	Reference	
HF	DM	1.50 (0.85-2.65)	0.885
	No DM	Reference	
No HF	DM	1.37 (0.79-2.38)	
	No DM	Reference	
CAD	DM	2.06 (1.08-3.94)	0.117
	No DM	Reference	
No CAD	DM	1.16 (0.68-1.97)	
	No DM	Reference	
PAD	DM	0.96 (0.10-9.76)	0.682
	No DM	Reference	
No PAD	DM	1.46 (0.98-2.19)	
	No DM	Reference	
Previous stroke	DM	1.17 (0.45-3.10)	0.924
	No DM	Reference	
No previous stroke	DM	1.47 (0.95-2.28)	
	No DM	Reference	
Rhythm control	DM	1.08 (0.34-3.44)	0.726
	No DM	Reference	
Rate control	DM	1.56 (1.03-2.37)	
	No DM	Reference	

Multivariable analysis adjusted for: age, sex, hypertension, heart failure, coronary artery disease, peripheral artery disease, and previous stroke.

Asia-Pacific Heart Rhythm Society Atrial Fibrillation Registry Investigators:

Hong Kong: Chun-Wah Siu David (Queen Mary Hospital).

Japan: Wataru Shimizu, Kenji Yodogawa (Department of Cardiovascular Medicine Graduate School of Medicine Nippon Medical School); Hiroyuki Tsutsui, Yasushi Mukai (Department of Cardiovascular Medicine, Faculty of Medical Sciences, Kyushu University); Hirofumi Tomita, Daisuke Horiuchi (Department of Cardiology, Hirosaki University Graduate School of School of Medicine); Joji Hagii (Hirosaki Stroke and Rehabilitation Center); Kazutaka Aonuma (Division of Cardiology, University of Tsukuba Hospital); Yasuo Okumura (Division of Cardiology, Nihon University Itabashi Hospital); Masahiko Goya, Kenzo Hirao (Department of Cardiovascular Medicine, Tokyo Medical and Dental University); Masayoshi Ajioka (Division of Cardiology, Tosei General Hospital); Nobuhisa Hagiwara, Atsushi Suzuki (Department of Cardiology, Tokyo Women's Medical University); Teiichi Yamane (Department of Cardiovascular Medicine, Jikei University); Takanori Ikeda, Hitomi Yuzawa (Toho University (Faculty of Medicine)); Kazuhiro Satomi, Yoshinao Yazaki (Heart Rhythm Center, Tokyo Medical University); Keiichi Fukuda(Department of Cardiology, Keio University School of Medicine); Yoshinori Kobayashi, Norishige Morita (Division of Cardiology, Tokai University Hachioji-hospital); Toyoaki Murohara (Department of Cardiology, Nagoya University); Eiichi Watanabe, Masahide Harada (Department of Cardiology, Fujita Health University School of Medicine); Satoru Sakagami, Takahiro Saeki (National Hospital Organization Kanazawa Medical Center); Kengo Kusano, Koji Miyamoto (Department of Cardiovascular Medicine, National Cerebral and Cardiovascular Center); Shinsuke Miyazaki, Hiroshi Tada (Department of Cardiovascular Medicine , Faculty of Medical Sciences, University of Fukui); Koichi Inoue, Nobuaki Tanaka(Cardiovascular center, Sakurabashi Watanabe Hospital); Yukihiro Koretsune, Haruhiko Abe (National Hospital Organization Osaka National Hospital, (Osaka, Japan); Yasuki Kihara, Yukiko Nakano (Department of Cardiovascular Medicine, Hiroshima University Graduate School of Biomedical and Health Sciences); Akihiko Shimizu, Yasuhiro Yoshiga (Department of Medicine and Clinical Science、University Graduate School of Medicine); Tomohiro Sakamoto, Ken Okumura(Division of Cardiology, Saiseikai Kumamoto Hospital Cardiovascular Center); Naohiko Takahashi, Tetsuji Shinohara (Oita University Hospital); Kyoko Soejima (Department of Cardiovascular Medicine, Kyorin University School of Medicine); Masahiko Takagi(Kansai Medical University Medical Center); Mitsuharu Kawamura, Yumi Munetsugu (Division of Cardiology, Showa University School of Medicine)

Korea: Sung-Hwan Kim (Division of Cardiology, Department of Internal Medicine, Seoul St. Mary's Hospital, College of Medicine, The Catholic University of Korea, Seoul, South Korea); Jae-Min Shim (Division of Cardiology, Korea University College of Medicine and Korea University Medical Center, Seoul, Republic of Korea); Jae Sun Uhm (Division of Cardiology, Department of Internal Medicine, Yongin Severance Hospital, Yonsei University College of Medicine, Yongin, Korea); Sung Il Im (Division of Cardiology, Department of Internal Medicine, Kosin University Gospel Hospital, Kosin University College

of Medicine, Busan, Korea); Hyoung-Seob Par (Division of Cardiology, Department of Internal Medicine, Keimyung University Dongsan Hospital, Daegu, South Korea); Jun Hyung Kim (Department of Cardiology, Chungnam National University, Daejeon, Republic of Korea); Young Keun On (Division of Cardiology, Department of Medicine, Heart Vascular and Stroke Institute, Samsung Medical Center, Sungkyunkwan University School of Medicine); Il-Young Oh (Division of Cardiology, Department of Internal Medicine, Seoul National University Bundang Hospital); Seung Yong Shin (Cardiovascular & Arrhythmia Centre, Chung-Ang University Hospital, Chung-Ang University, Seoul, Korea); Jum Suk Ko (Division of Cardiology, Department of Internal Medicine, Wonkwang University School of Medicine, Iksan, Korea); Jun Beom Park (Department of Cardiology, College of Medicine, Ewha Womans University, Seoul, Korea)

Singapore: Wee-Siong Teo (National Heart Centre Singapore); Kelvin Cheok-Keng Wong (Changi General Hospital); Toon-Wei Lim (National University Hospital); David Foo (Tan Tock Seng Hospital)

Taiwan: Shih-Ann Chen (Taichung Veterans General Hospital); Shih-Ann Chen, Tze-Fan Chao, Yenn-Jiang Lin, Fa-Po Chung, Yu-Feng Hu, Shil-Lin Chang, Ta-Chuan Tuan, Jo-Nan Liao (Taipei Veterans General Hospital); Cheng-Hung Li, Jin-Long Huang, Yu-Cheng Hsieh, Tsu-Juey Wu, Ying-Chieh Liao (Taichung Veterans General Hospital); Cheng-Hung Chiang, Hsiang-Chiang Hsiao, Tung-Chen Yeh (Kaohsiung Veterans General Hospital); Wei-Siang Lin, Wen-Yu Lin (Tri-Service General Hospital); Jen-Yuan Kuo, Chong-Lie Hong, Yih-Je Wu, Ying-Siang Li, Jui-Peng Tsai, Kuo-Tzu Sung, Sheng-Hsiung Chang (Mackay Memorial Hospital).
